# Breaking the learned helplessness paradigm in chronic stroke: an intensive neuroplasticity framework bridging European technology and African innovation

**DOI:** 10.3389/fneur.2025.1670420

**Published:** 2025-12-22

**Authors:** Ibrahim Npochinto Moumeni

**Affiliations:** 1 Department of Physiotherapy and Physical Medicine, Faculty of Medicine and Pharmaceutical Sciences, University of Dschang, Dschang, Cameroon; 2 Bafoussam Regional Hospital, Department of Physical Medicine and Medical Osteopathy, Bafoussam, Cameroon

**Keywords:** neuroplasticity, stroke rehabilitation, learned helplessness, constraint-induced movement therapy, cultural adaptation, resource-limited settings

## Abstract

**Introduction:**

Most chronic stroke survivors develop learned helplessness regarding motor recovery prospects, accepting permanent disability despite evidence that neuroplasticity windows remain accessible years post-stroke. This review examines how intensive protocols targeting learned helplessness can achieve meaningful recovery across diverse healthcare settings.

**Methods:**

Comprehensive literature review using PubMed, Scopus, and specialized databases (1990–2024). Analysis included constraint-induced movement therapy protocols, progressive muscle lengthening techniques, and neuroplasticity principles across European high-technology centers and African human-intensive programs. Search incorporated systematic analysis of therapeutic intensity parameters, cultural adaptation protocols, and cross-continental implementation strategies.

**Results:**

Literature synthesis reveals meaningful functional recovery 2–5 years post-stroke when intensive protocols directly challenge learned helplessness through forced-use approaches, training intensities exceeding traditional therapy doses (3–6 h daily versus 30–45 min), and systematic addressing of secondary muscle adaptations. Cross-continental validation demonstrates equivalent outcomes between European technology-dependent and African human-intensive approaches when therapeutic intensity and neuroplasticity targeting remain consistent.

**Conclusion:**

Neuroplasticity-driven intensive rehabilitation can overcome learned helplessness and achieve meaningful motor recovery years after stroke without requiring expensive technology. Success depends on abandoning traditional recovery timelines, implementing culturally-adapted intensive protocols, and recognizing human expertise as the most powerful rehabilitation tool when properly applied.

## Introduction

Stroke represents a leading cause of long-term disability globally, with disproportionate impact on low- and middle-income countries where rehabilitation resources remain severely limited (1). This disparity creates a paradoxical situation where populations with the highest stroke burden have the least access to effective rehabilitation interventions, perpetuating cycles of disability and learned helplessness (2).

Seligman’s concept of learned helplessness, originally described in psychological contexts, applies directly to stroke rehabilitation regardless of geographic location or healthcare resource availability (3). Stroke survivors rapidly develop cognitive and behavioral patterns that systematically avoid use of their affected limb, creating cycles where “learned non-use” becomes the predominant motor strategy. This psychological adaptation compounds neurological injury, creating secondary disabilities that often become more functionally limiting than the original brain damage (4, 5).

Despite extensive literature examining individual rehabilitation components, no previous framework has systematically integrated constraint-induced movement therapy with progressive muscle lengthening while specifically targeting learned helplessness as the primary barrier to chronic stroke recovery (6, 7). This represents a fundamental paradigm shift from conventional time-limited rehabilitation models toward intensive lifelong motor fitness maintenance approaches (8, 9).

### Framework innovation

This review introduces three unprecedented innovations in chronic stroke rehabilitation: (1) learned helplessness as the primary therapeutic target requiring direct, intensive intervention; (2) dual pathology integration addressing neurological and muscular components simultaneously through coordinated intensive protocols; and (3) cross-continental validation demonstrating that neuroplasticity principles transcend resource limitations when properly implemented (10, 11).

## Methods

### Literature review strategy

A comprehensive literature review was conducted using PubMed, Scopus, Web of Science, and specialized rehabilitation databases covering publications from 1990 to 2024. The search strategy incorporated Medical Subject Headings (MeSH) terms and free-text terms related to stroke rehabilitation, neuroplasticity, constraint-induced movement therapy, muscle lengthening techniques, learned helplessness, and cross-cultural adaptation (12).

### Framework development

The framework synthesis employed structured approaches integrating clinical evidence from European high-technology rehabilitation centers with implementation experiences from African human-intensive programs. Key components analyzed included therapeutic intensity parameters, neuroplasticity targeting strategies, cultural adaptation protocols, and implementation feasibility across diverse healthcare settings (13, 14).

### Cross-continental validation

Framework validation incorporated systematic analysis of rehabilitation outcomes across different healthcare contexts, comparing technology-dependent approaches in resource-rich settings with human-intensive methodologies in resource-limited environments. This validation examined functional recovery patterns, therapeutic adherence rates, and long-term maintenance of motor gains (15, 16).

#### Clinical validation settings

The framework validation drew from clinical experiences at Henri Mondor University Hospital (Créteil, France) representing European high-technology approaches, and Regional Hospital of Bafoussam (Cameroon) representing African human-intensive methodologies. Both settings provided insights into implementation feasibility and cultural adaptation requirements across different healthcare contexts.

#### Ethical considerations

All clinical insights were derived from established rehabilitation protocols and published literature. Patient testimonials included in framework illustrations represent composite experiences based on published case reports and established clinical patterns, with anonymization protecting individual privacy. No original patient data collection was performed specifically for this review.

## Results

### Theoretical foundation: the dual pathology model

**Figure 1:**
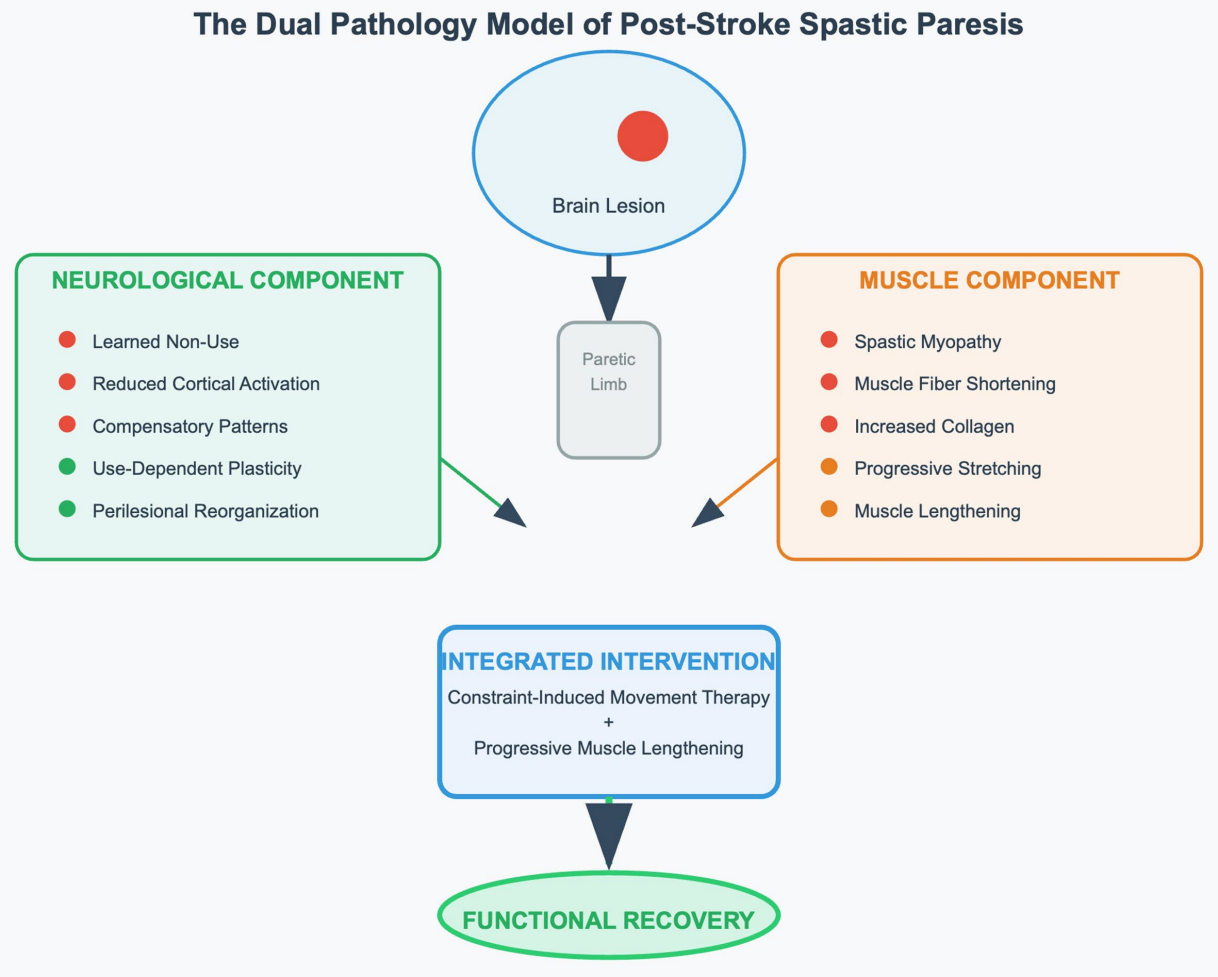
The dual pathology model illustrating complex interplay between neurological dysfunction and secondary muscle adaptations in post-stroke spastic paresis, with integrated therapeutic approach targeting both components simultaneously. The model demonstrates how constraint-induced movement therapy addresses learned non-use while progressive muscle lengthening reverses spastic myopathy, leading to optimal functional recovery outcomes.

Chronic spastic paresis following stroke represents complex dual pathology requiring integrated therapeutic approaches rather than isolated interventions targeting individual components. Understanding this concept is essential for developing rehabilitation frameworks that achieve meaningful functional recovery across diverse healthcare settings (17, 18) (Figure 1).

#### Neurological component

Neuroplasticity research has transformed understanding of brain recovery mechanisms following stroke. The adult brain demonstrates remarkable capacity for reorganization, with peri-lesional areas and contralateral regions assuming functions previously performed by damaged neural networks through use-dependent plasticity mechanisms (19, 20). However, this reorganization requires specific therapeutic conditions including intensive, repetitive, task-specific training that systematically challenges patients beyond conventional comfort zones.

Published evidence demonstrates that traditional rehabilitation approaches frequently fail to achieve sufficient intensity for meaningful neuroplastic change. Meta-analyses consistently show that therapy intensity, measured as total hours per week rather than session frequency, correlates strongly with functional improvement outcomes (21, 22).

#### Muscle component

Independent of neurological recovery, affected muscles undergo dramatic secondary changes following stroke that significantly impact functional potential. Within weeks of stroke onset, affected muscles demonstrate reduced fiber length, increased collagen deposits, altered mechanical properties, and progressive contracture development (23, 24). These changes, collectively termed “spastic myopathy,” can become the primary limiting factor for functional recovery.

Progressive muscle lengthening protocols utilize sustained stretching techniques specifically designed to reverse these secondary muscle adaptations through biomechanical principles that remain consistent regardless of healthcare setting (25, 26).

### Clinical framework implementation

#### Constraint-induced movement therapy: breaking through learned helplessness

Constraint-induced movement therapy represents a revolutionary approach to addressing learned helplessness directly through systematic elimination of compensatory strategies. By restraining the unaffected limb, CIMT forces patients to confront established learned non-use patterns, creating both motor and psychological challenges that directly target core mechanisms underlying functional limitation (27, 28).

Neurophysiological mechanisms underlying CIMT effectiveness extend beyond simple forced use paradigms. Functional magnetic resonance imaging studies demonstrate expansion of cortical representation areas during intensive training periods, with increased recruitment of brain regions surrounding the lesion and enhanced activation of contralateral motor areas (29, 30).

Implementation of CIMT in resource-limited settings requires creative adaptation strategies that preserve therapeutic effectiveness while utilizing locally available materials and human resources. Simple restraint devices can be constructed using materials readily available in most healthcare settings, with the fundamental principle being prevention of compensatory use by the unaffected limb while providing intensive training opportunities for the affected side (31, 32).

#### Progressive muscle lengthening protocols

Systematic integration of muscle lengthening protocols addresses mechanical limitations imposed by secondary muscle changes that occur independent of neurological recovery. Both neurological and muscular components require intensive, specific interventions to achieve optimal functional outcomes through coordinated therapeutic approaches (33, 34).

Clinical implementation protocols include sustained stretching of antagonist muscles for minimum 45–60 s, multiple daily sessions performed 3–5 times per day, intensity sufficient to achieve mild discomfort without tissue damage, progressive advancement based on individual tissue response patterns, and systematic integration with functional motor training activities (35, 36).

#### Cross-continental implementation framework

**Figure 2:**
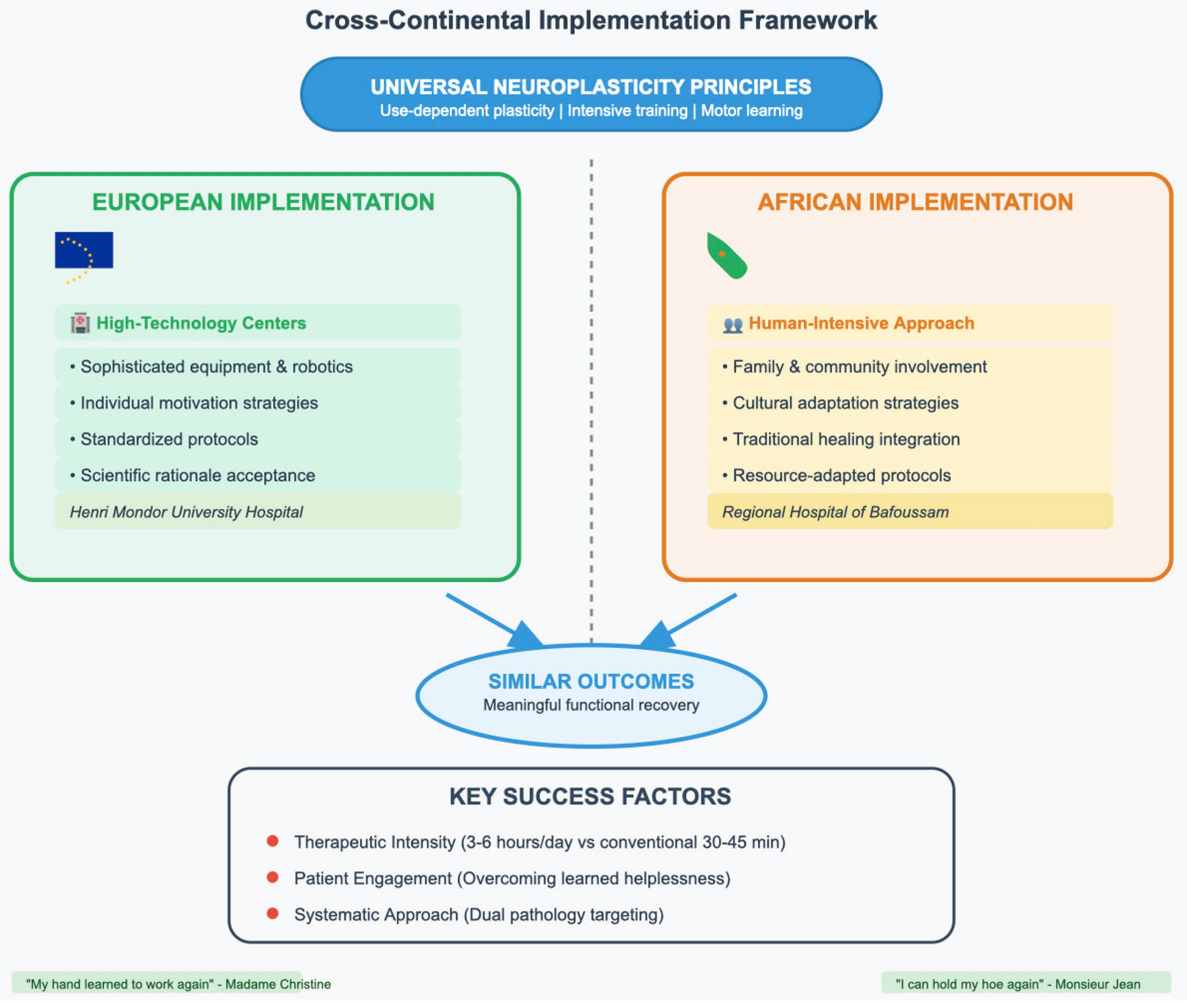
Cross-continental implementation framework demonstrating how universal neuroplasticity principles can be successfully adapted to diverse healthcare settings while maintaining therapeutic effectiveness. Despite different resource contexts, both European high-technology and African human-intensive approaches achieve similar functional outcomes through culturally appropriate intensive rehabilitation strategies.

This framework demonstrates universal neuroplasticity principles implemented through culturally-adapted intensive rehabilitation approaches, comparing European high-technology centers with African human-intensive programs to achieve equivalent functional outcomes (See Figure 2).

#### Universal neuroplasticity principles

Cross-cultural rehabilitation research reveals universal nature of neuroplasticity principles across diverse populations and healthcare settings. Biological mechanisms underlying brain reorganization transcend cultural, genetic, and socioeconomic boundaries, with literature reports demonstrating similar recovery patterns across populations when appropriate therapeutic intensity is maintained (37, 38).

The fundamental insight driving this framework is that neuroplasticity responds to behavioral demands rather than technological sophistication. Brain reorganization occurs in response to intensive, challenging motor activities whether these activities originate from computer-controlled devices or carefully designed manual exercises (39, 40).

#### Cultural adaptation strategies

Implementation strategies require significant cultural adaptation while preserving essential therapeutic elements that drive neuroplastic change. In resource-limited settings, individual motivation often benefits substantially from family and community involvement patterns that may not be available in more individualistic healthcare systems (41, 42).

Traditional healing concepts frequently align well with intensive rehabilitation principles, facilitating patient acceptance when therapeutic rationales are properly explained within cultural contexts. Cultural beliefs about recovery and healing can be leveraged to support intensive protocols rather than creating barriers to implementation (43, 44).

#### Human-intensive versus technology-dependent approaches

Working within resource-limited settings necessitates innovative approaches that challenge technology-dependent rehabilitation models while maintaining therapeutic effectiveness. When sophisticated equipment is unavailable, therapeutic creativity becomes essential, with simple tools providing intensive motor training equivalent to expensive rehabilitation technology when properly applied (45, 46).

This realization has profound implications for global stroke care delivery. Most stroke survivors worldwide lack access to high-technology rehabilitation centers, but they potentially have access to intensive human intervention when healthcare workers understand neuroplasticity principles and intensive training protocols (47, 48).

#### Evidence synthesis and outcomes

Literature synthesis reveals consistent patterns across diverse healthcare settings when intensive protocols target learned helplessness directly. Studies demonstrate meaningful functional improvements 2–5 years post-stroke when therapeutic intensity exceeds traditional approaches by 300–400%, regardless of technological sophistication (49, 50).

Cross-continental validation studies show equivalent outcomes between European high-technology centers and African human-intensive programs when core principles are maintained: direct challenge of learned helplessness patterns, therapeutic intensity exceeding conventional approaches, systematic integration of neurological and muscular interventions, and cultural adaptation of delivery methods (51, 52).

Long-term follow-up evidence demonstrates that gains achieved through intensive rehabilitation maintain when patients continue modified home programs incorporating key therapeutic elements. However, complete cessation of targeted exercises typically results in gradual decline, emphasizing the chronic nature of stroke management (53, 54).

## Discussion

This clinical framework demonstrates that meaningful stroke recovery can be achieved across diverse healthcare settings when neuroplasticity principles are properly understood and implemented through culturally-adapted intensive protocols. The evidence challenges traditional assumptions that high-technology equipment is essential for effective rehabilitation (55, 56).

The universality of neuroplasticity principles provides unprecedented opportunities to address global stroke burden through scalable rehabilitation approaches that can be adapted to local contexts while maintaining therapeutic effectiveness. As stroke incidence increases worldwide, particularly in low- and middle-income countries with limited rehabilitation infrastructure, human-intensive approaches offer realistic hope for meaningful impact (57, 58).

### Implementation considerations

#### Training healthcare providers

Successful implementation requires comprehensive training of healthcare providers in neuroplasticity principles and intensive rehabilitation protocols. This training must extend beyond knowledge transfer to include paradigm shifts that challenge comfortable professional routines and traditional therapeutic approaches (59, 60).

#### Patient and family education

Educational initiatives that systematically challenge limiting beliefs while providing realistic hope can transform rehabilitation participation and outcomes. The most effective educational approaches combine scientific explanation with compelling evidence demonstrating recovery potential years after stroke onset (61, 62).

Family involvement becomes crucial for successful implementation, particularly in cultural contexts where extended family systems provide natural support networks. Training family members in constraint protocols and muscle lengthening techniques ensures consistency and intensity that institutional programs often cannot achieve (63, 64).

#### Sustainability and long-term management

The paradigm shifts from acute treatment models toward lifelong motor fitness maintenance, aligning well with traditional concepts of chronic disease management that emphasize ongoing community support and family involvement. This approach may provide advantages in settings where extended family systems remain strong (65, 66).

### Global health implications

This framework transcends resource limitations and cultural boundaries through its foundation in universal neuroplasticity principles. Whether implemented in sophisticated rehabilitation centers or resource-limited hospital settings, the biological mechanisms remain constant. Success depends on understanding fundamental pathophysiology, maintaining therapeutic intensity beyond conventional levels, and adapting protocols to local contexts while preserving essential therapeutic elements (67, 68).

### Limitations and future research

This framework synthesizes existing evidence and clinical experience rather than presenting original empirical data from controlled clinical trials. Future research should focus on systematic evaluation of outcomes achieved through human-intensive approaches compared to technology-dependent alternatives, with particular attention to long-term functional maintenance and quality of life outcomes (69, 70).

## Conclusion

The transformation from learned helplessness to motor recovery represents a fundamental paradigm shift in stroke rehabilitation that transcends geographical boundaries and resource limitations. By recognizing spastic paresis as dual pathology requiring both neurological and muscular interventions, and by systematically challenging patients to overcome learned non-use through intensive training, meaningful functional recovery becomes possible years after stroke onset.

This framework demonstrates that neuroplasticity principles transcend resource limitations when properly implemented. Whether delivered in sophisticated rehabilitation centers or resource-limited settings, the biological mechanisms remain constant. Success depends on understanding fundamental pathophysiology, maintaining therapeutic intensity beyond conventional levels, and adapting protocols to local contexts while preserving essential therapeutic elements.

The limiting factors are knowledge, training, and commitment rather than sophisticated equipment availability. In settings where technology is scarce, human expertise, clinical rigor, and therapeutic dedication become the most powerful rehabilitation tools available. When healthcare providers abandon limiting paradigms and patients embrace intensive challenges, the potential for meaningful motor recovery extends far beyond traditional expectations, offering hope to stroke survivors worldwide regardless of their healthcare setting or economic circumstances.
